# Bilateral acute tibial osteomyelitis in a patient without an underlying disease: a case report

**DOI:** 10.1186/1752-1947-8-388

**Published:** 2014-11-26

**Authors:** Serkan Sipahioglu, Huseyin Askar, Sinan Zehir

**Affiliations:** 5 Department of Orthopedics and Traumatology, Harran University Medical Faculty, Yenisehir Street, 63000 Sanliurfa, Turkey; 6 Department of Orthopedics and Traumatology, Samandag Ministry of Health Hospital, Samandag, 31800 Hatay Turkey; 7 Department of Orthopedics and Traumatology, Hitit University Medical Faculty, Camlik Street, 19000 Corum, Turkey

**Keywords:** Child, Chronic disease, Osteomyelitis, Tibia

## Abstract

**Introduction:**

The simultaneous presentation of osteomyelitis in more than one bone is rare and is commonly accompanied by a chronic disease. Even in such cases, other conditions that arise commonly in the long bones of children—such as chronic recurrent multifocal osteomyelitis and Ewing’s sarcoma—must be ruled out.

**Case presentation:**

We present the case of a 5-year-old boy with bilateral acute tibial osteomyelitis without an underlying chronic disease who was treated with surgical debridement. We also review the pertinent literature.

**Conclusion:**

Early diagnosis, appropriate antibiotic therapy, and timely surgical intervention—including proactive efforts to prevent fractures—all increase the chance of a successful outcome for these patients.

## Introduction

Acute hematogenous osteomyelitis is the most common type of bone infection. The annual incidence of this infection is 1:5000 children younger than 13 years of age, and most of them are younger than 5 years of age. It arises characteristically in the metaphysis of long bones, such as the femur, tibia, and humerus [1]. Surgery followed by antibiotic therapy is a suitable approach for patients who do not respond to parenteral antibiotics [1]. The recommended duration of antibiotic therapy is 4 to 8 weeks, although successful outcomes have been reported in uncomplicated cases with a mean of only 23 days of therapy [2]. The simultaneous involvement of two or more bones has rarely been reported in the literature. When it does occur, it is usually accompanied by an underlying chronic disease, such as sickle cell anemia [3]. In this report, we present a case of osteomyelitis with bilateral tibial involvement that developed in a child without a chronic disease or preceding trauma. Our report is highlighted by a discussion of the differential diagnosis.

## Case presentation

**Figure 1:**
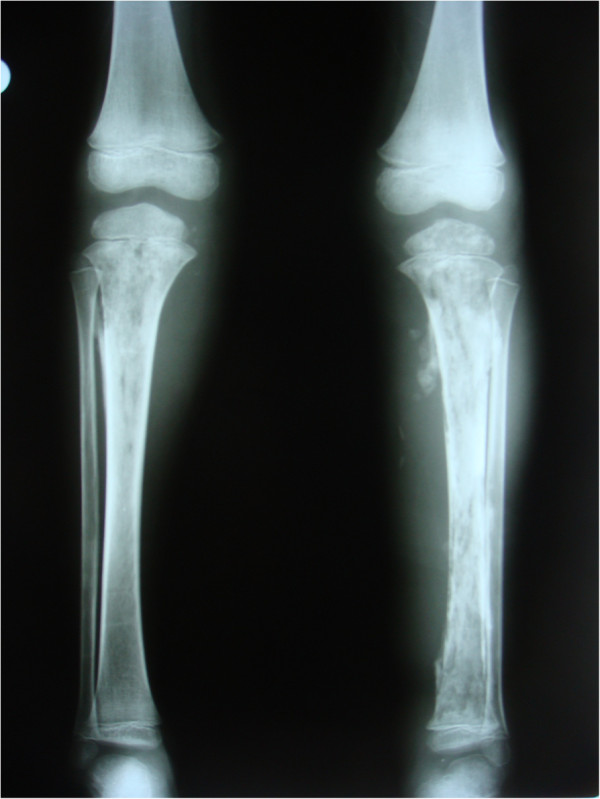
**Anteroposterior radiograph of both tibia showing periosteal reactions and cortical destruction.**

A 5-year-old boy presented to our institution with a complaint of pain, swelling, and redness in both legs. His parents stated that they had taken him to another health institution 40 days earlier because he had a fever and that a 10-day course of oral antibiotic therapy was prescribed. During a physical examination at our institution, swelling, erythema, and warmth were detected in both legs, and a purulent discharge was observed exuding from the proximal end of each tibia. He did not have a chronic illness or a history of a trauma. His laboratory tests revealed a white blood cell count of 15,640/mm^3^, C-reactive protein of 5.29μg/mL, and erythrocyte sedimentation rate of 63mm/h. The results of a Brucella tube agglutination test, Gruber Widal test (for *Salmonella*), and purified protein derivative test for tuberculosis (TB) (the TB test was previously done twice and was negative each time). The patient did not complain of a cough or fever, and his chest X-ray was normal. X-rays of his legs revealed cortical destruction and an increase in periosteal tissue in the proximal aspect of each tibia and the distal aspect of the left tibia (Figure 1). Additionally, magnetic resonance imaging revealed bone marrow edema throughout both tibias, an accumulation of purulent debris in the bone marrow and metaphysis, and pyomyositis contiguous with the site of osteomyelitis. On radionuclide imaging studies, we found an increased uptake of technetium at the site of infection (that is, in the metaphysis of each tibia).

After diagnosing his condition, we carried out open irrigation and surgical drainage in his legs. After opening cortical fenestrations in the metaphysis of each tibia, we performed curettage of the medullary canal, carefully avoiding the growth plate. Approximately 150cc of purulent fluid was drained from the left tibia and approximately 200cc from the right tibia. Microbiological and pathological samples were taken during surgery for Gram staining, blood and bone cultures, staining for acid-resistant bacilli, and a TB culture, all of which were negative. The results of a pathological examination were consistent with acute osteomyelitis. In our search for an underlying chronic disease, we carried out the following tests, all of which produced no indications of any abnormalities: urinalysis; a routine biochemical analysis; a peripheral blood smear; hemoglobin electrophoresis (to rule out sickle cell anemia); an abdominal ultrasound; immunoglobulin and complement factor analysis; tests for rheumatoid factor, antinuclear antibody, anti-Smith antibody, and anti-ribonucleoprotein antibody; intermittent *Salmonella* agglutination tests; VDRL; and urine and blood cultures.

**Figure 2:**
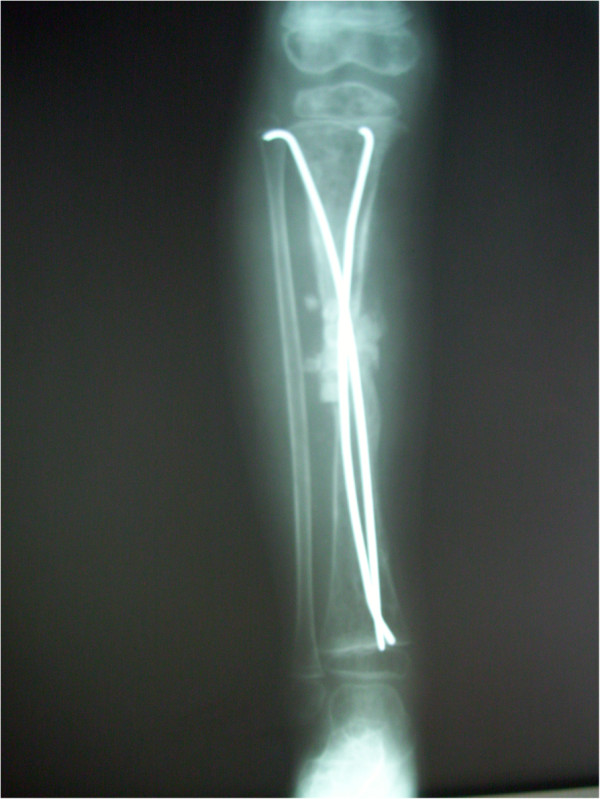
**Anteroposterior and lateral radiographs of the patient’s left tibia after intramedullary nailing done for pathologic fracture.**

**Figure 3:**
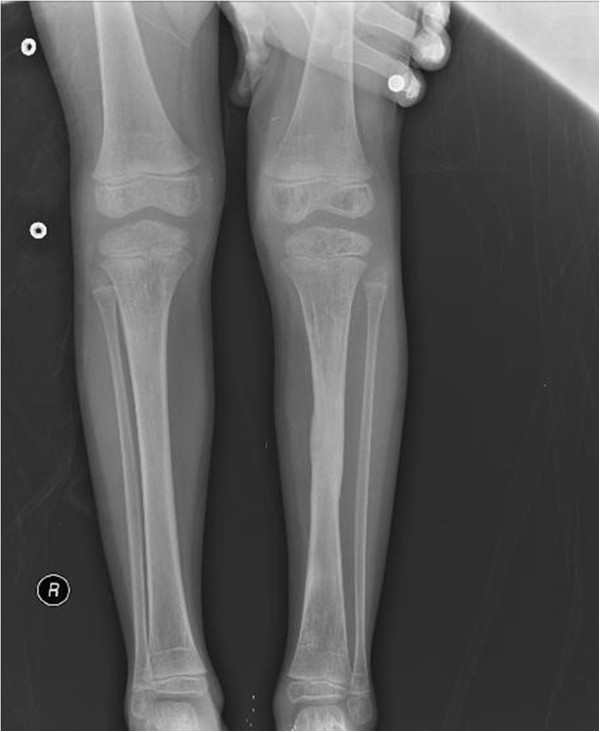
**Healed fracture and osteomyelitis at the 18th month of treatment.**

After surgery, we initiated empiric parenteral antibiotic therapy using ampicillin-sulbactam and gentamicin following the recommendations of the Pediatric Infectious Disease Department. Because all cultures were negative, we continued this therapy for three weeks. During that time, the laboratory test results and the patient’s physical condition showed clear signs of improvement. At the end of the third week of therapy, we changed the antibiotic to amoxicillin-clavulanate and the route of administration to oral. At the end of the fourth week of therapy, the splints were removed from the patient’s legs, and protected weight-bearing was initiated.Three months later, the boy developed a fracture in the left tibia along the fenestration that had been opened for drainage. A titanium elastic nail was used for fixation, and the lesion was completely debrided during surgery (Figure 2). Defects that developed after the debridement procedure were corrected using corticocancellous grafting. Tissue cultures, biopsy, and Gram staining were carried out, all with negative results. At the end of the sixth week, the splints were removed from the patient’s legs and protected weight-bearing was initiated. The follow-up period passed without incident, and all signs of infection disappeared. The fracture was completely healed within 1 year, at which time we removed the titanium elastic nail. All blood test and radiographic findings were normal at the 18-month follow-up visit (Figure 3).

## Discussion

Osteomyelitis is rarely seen in more than one bone at the same time. When it does appear simultaneously in two or more bones, it is usually accompanied by a chronic illness, such as sickle cell anemia. For example, Bachmeyer and colleagues reported bilateral tibial osteomyelitis caused by *Pantoea agglomerans* (a rare gram-negative bacillus) in a patient with sickle cell anemia [3]. Other disorders seen with bilateral tibial osteomyelitis include Guillain–Barré syndrome, retroperitoneal fibrosis, and systemic lupus erythematosus. Javaloyas and colleagues treated two patients who developed bilateral symmetric osteomyelitis caused by a Gram-negative bacillus after a latent period following an episode of sepsis [4]. Picillo and colleagues reported the case of a patient with systemic lupus erythematosus and arthritis in the knee, accompanying bilateral femoral septic osteomyelitis caused by *Salmonella enteritidis*[5]. In some cases, an underlying condition is not found. Crook, for example, carried out numerous tests to search for an underlying condition in a patient with bilateral tibial osteomyelitis caused by *Staphylococcus aureus*, but every test was negative [6]. Similarly, we failed to identify any underlying chronic disorders in our patient, other than anemia, and his hemoglobin level (the basis of the diagnosis) returned to normal after treatment. At his 18-month follow-up visit, we found no new signs or symptoms and concluded that he did not have any other disease.

Chronic recurrent multifocal osteomyelitis is another disorder that needs to be ruled out in the differential diagnosis of bilateral osteomyelitis. King and colleagues proposed the following diagnostic criteria: two or more bone lesions identified radiographically, pain and swelling lasting at least six months (including periods of remission), and failure to respond to antibiotic therapy given for at least one month [7]. Chronic recurrent multifocal osteomyelitis is usually accompanied by other inflammatory conditions, such as palmoplantar pustulosis, psoriasis, inflammatory bowel disease, and acne. A biopsy often reveals acute and chronic inflammation with lymphocyte dominance, but cultures are almost always negative. The pathogenesis of this condition remains unknown, although an autoinflammatory process or an infection by an unrecognized causative agent has been suggested [8]. Because our patient responded to antibiotic therapy and purulent material was drained from both tibias, we were able to exclude this diagnosis.

Ewing’s sarcoma—the second most common primary malignant tumor of bone in children—should also be ruled out. Ewing’s sarcoma has a predilection for individuals ages 10 to 20 years. Its initial symptoms may include pain and swelling. X-rays reveal radiolucent areas suggestive of a periosteal reaction that sometimes can be distinguished from osteomyelitis only by histopathological examination [9]. *Mycobacterium* tuberculosis, which can have signs of extrapulmonary involvement, may also cause an osteoarticular infection, although this is not common [10]. Tuberculous osteomyelitis can be diagnosed by isolation of *Mycobacterium* tuberculosis from cultures of bone biopsy material. A positive tuberculin skin test, Ziehl–Neelsen staining, polymerase chain reaction assays, radiological findings, and histopathology of the bone lesions are also helpful in confirming the diagnosis. We were able to exclude both TB and Ewing’s sarcoma on the basis of laboratory and histopathological findings.

## Conclusions

The simultaneous presentation of osteomyelitis in more than one bone is rare and is commonly accompanied by a chronic disease. As we observed in our patient, it can also develop in the absence of an underlying disease. Even in such cases, other conditions that arise commonly in the long bones of children must be ruled out. Early diagnosis, appropriate antibiotic therapy, and timely surgical intervention all increase the chance of a successful outcome for these patients.

## Consent

Written informed consent was obtained from the patient’s father for publication of this case report and accompanying images. A copy of the written consent is available for review by the Editor-in-Chief of this journal.
